# Validation of the psychometric properties of the Malay advance care planning questionnaire

**DOI:** 10.1186/s12904-021-00790-7

**Published:** 2021-07-15

**Authors:** Mun Kit Lim, Pauline Siew Mei Lai, Pei Se Wong, Sajaratulnisah Othman, Fadzilah Hanum Mohd Mydin

**Affiliations:** 1 School of Pharmacy, Monash University Malaysia, Jalan Lagoon Selatan, Bandar Sunway, 47500 Selangor Darul Ehsan Malaysia; 2 Department of Primary Care Medicine, Faculty of Medicine, University of Malaya, Jalan Profesor Diraja Ungku Aziz, 50603 Wilayah Persekutuan Kuala Lumpur, Malaysia; 3 School of Pharmacy, International Medical University, Jalan Jalil Perkasa 19, Wilayah Persekutuan Kuala Lumpur, 57000 Malaysia

**Keywords:** Advance care planning, Psychometric properties, Validation, Malaysia

## Abstract

**Background:**

There is a growing interest among the developing countries on advance care planning (ACP) due to the reported benefits of planning ahead in the developed countries. Validated instruments in various languages have been developed to facilitate study on the views of public prior to its implementation. However, instrument to explore the views on ACP in Malay has not been developed and validated yet, even though Malay is spoken extensively by approximately 220 million people in the Malay Archipelago. There is also a need for instrument in Malay language to facilitate the assessment of knowledge, attitude and practice (KAP) of Malaysians regarding ACP. Therefore, the aim of this study was to validate the psychometric properties of the Malay Advance Care Planning Questionnaire (ACPQ-M).

**Methods:**

The ACPQ was translated according to international guidelines. This validation study was conducted from January to June 2018. Participants who were ≥ 21 years old, and able to understand Malay were recruited from an urban primary care clinic and a tertiary education institution in Malaysia. A researcher administered the ACPQ-M to participants via a face-to-face interview at baseline and 2 weeks later. Each interview took approximately 10–20 min.

**Results:**

A total of 222/232 participants agreed to participate (response rate = 96.0%). Exploratory factor analysis and confirmatory factor analysis found that the ACPQ-M was a 4-factor model. The Cronbach’s α values for the four domains ranged from 0.674–0.947. Only 157/222 participants completed the test-retest (response rate = 71%). At test-retest, quadratic weighted kappa values for all domains ranged from 0.340–0.674, except for two domains which ranged from − 0.200-0.467.

**Conclusions:**

The ACPQ-M was found to be a 4-factor model, and a valid and reliable instrument to assess the KAP regarding ACP. This instrument can contribute to profound understanding of the KAP of Malaysians regarding ACP, and assist policy makers in determining the readiness for legislation of ACP in Malaysia.

## Background

Advance care planning (ACP) is a process which supports a person at various stages of life and health status to understand and express their values, wishes and preferences toward future health care [1]. Patients can record their preference of medical care and appoint a surrogate decision-maker ahead of time [2]. Well implemented ACP policies have improved the quality of life of patients and their families [3, 4]. This improvement may be contributed by the enhancement of patients’ sense of control over their medical care and the bonding between patients, families and health care professionals with ACP discussions [5, 6]. Despite the known benefits, the uptake of ACP remains low [7] in countries which has legislation support such as the United States [8], United Kingdom [8] and Australia [9].

ACP is currently not legislated in Malaysia although it is briefly described in the code of medical ethics [10]. A study revealed that 76% of 56 patients undergoing haemodialysis in Malaysia have never heard of ACP [11]. Another exploratory study performed on fifteen older Malaysians found that they were receptive to its concept, although they had no prior exposure to ACP [12]. However, no in-depth work had been conducted in Malaysia to obtain further insights into their knowledge, attitude and practice (KAP) towards advance care planning. Moreover, recent studies on younger adults have highlighted the needs to engage younger adults in ACP [13, 14] as previous studies [15–19] were mainly conducted on adults aged > 50 years with terminal illnesses or chronic diseases. Hence, there is a need to include younger people when exploring the KAP regarding ACP. A search of published literature revealed that there is a lack of instruments that assessed the KAP regarding ACP. Previous KAP studies that were published used non-validated questionnaires [20, 21]. Instruments should be validated to ensure consistency and reliability of the results obtained [22].

Several instruments have been developed to assess the views, knowledge, attitude and practice of ACP: the ‘Cultural values and belief scale’ [16], ‘The ethnicity and attitudes toward advance care directives questionnaires’ [15] and the ‘Asian American quality of life survey’ [23]. These instruments were primarily tested and used in the United States [23], Korea [24], Japan [25] and Hong Kong [26]. Recognition of the influence of culture and religion on individual’s preference for advance care planning has highlighted the need to consider a local instrument for assessment of KAP regarding ACP among the Malaysians [27].

The English version of the Advance Care Planning Questionnaire (ACPQ) was developed and validated to assess the KAP on ACP in Malaysia [28]. This instrument was found to be a reliable instrument with adequate internal consistency to measure the four domains related to ACP; “feelings regarding ACP”, “justifications for ACP”, “justifications for not having ACP: fate and religion” and “justifications for not having ACP: avoid thinking about death”. However, Malay language is extensively use in the malay archipelago especially in Malaysia, Brunei, and Indonesia, and spoken by approximately 220 million people [29]. Therefore, this study translated the English Advance Care Planning Questionnaire (ACPQ) into Malay and aimed to validate the psychometric properties of the Malay Advance Care Planning Questionnaire (ACPQ-M).

## Methods

Validation of the ACPQ-M consists of two parts: translation of the validated English Advance Care Planning Questionnaire (ACPQ) to Malay, and its validation and reliability testing.

### Translation of the advance care planning questionnaire (ACPQ)

**Fig. 1:**
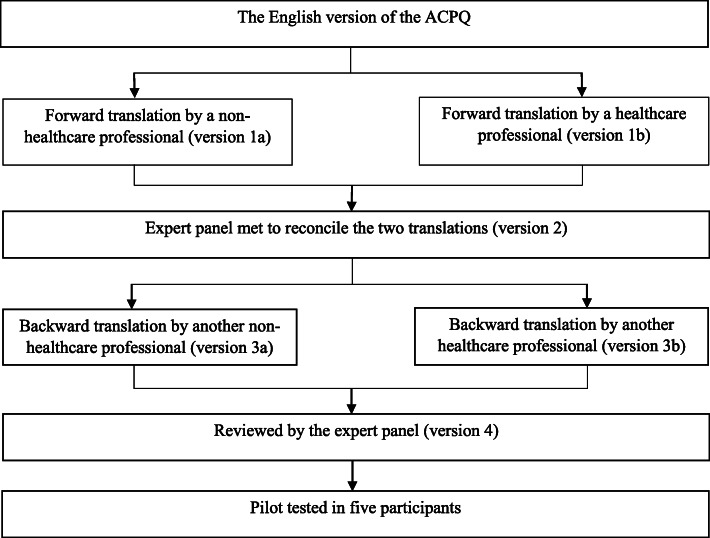
Translation of the Advance Care Planning Questionnaire (ACPQ) from English to Malay

The final version of the English ACPQ was reduced to 60 items from 66 items after exploratory factor analysis in the previous study [28]. Approval to use the English ACPQ was obtained from the copyright holder, who is the corresponding author in this study. The English ACPQ was translated according to the Principles of Good Practice for the Translation and Cultural Adaptation Process (Fig. 1) [30]. No problems were encountered during the pilot test. Hence, version 4 of the ACPQ-M was used.

### Validation of psychometric properties of the Malay advance care planning questionnaire (ACPQ-M)

#### Study design and setting

This validation study was conducted from January to June 2018 at an urban primary care clinic and a tertiary education institution in Malaysia.

#### Participants

The aim of our study was to validate the ACPQ-M among community dwellers i.e. ambulatory care patients as well as their family members, regardless of their health status; as the second phase of our study was to assess the readiness of community dwelling adults for ACP. Ambulatory care patients as well as their family members (i.e. community dwellers) who were ≥ 21 years and able to understand Malay were recruited using convenience sampling. Community dwellers with mental illnesses such as dementia or psychosis were excluded.

#### Sample size calculation

The sample size required to perform factor analysis was calculated based on rule of thumb of 10 participants per item [31, 32]. The number of items in the ACPQ that could be validated was 16 as the items had a 5-point Likert-scale response. Therefore, the total number of participants required was 16*10 = 160 participants. Accounting for a drop-out rate of 40% at retest [28], an additional 64 participants were recruited, bringing the total to 224 participants.

#### Instruments used

##### The Malay advance care planning questionnaire (ACPQ-M)

The ACPQ-M consists of four sections and 60 items (29 items were measure on nominal scale, whilst 31 items were measured on a 5-point Likert scale). Participants were required to answer all the items in section A, B and C of the ACPQ-M. Section A and B recorded the demographic and health information while section C focused on the knowledge of ACP [28]. As for section D of the ACPQ-M, those who were in in favour of ACP were asked to answer the items in the domain “justifications for ACP”; while those who were not in favour of ACP were asked to answer the items in the domain “justifications for not having ACP: avoid thinking about death” and “justifications for not having ACP: fate and religion”.

##### Procedure

Eligible participants were approached, and the purpose of the study was explained to them using the participant information sheet. Written informed consent was obtained from those who agreed to participate. A researcher administered the ACPQ-M to participants via a face-to-face interview as this questionnaire contained some medical terms which may not be understood by the lay person. Each interview took between 10 to 20 min. The retest was performed 2 weeks later over the phone.

##### Data analyses

Data analyses were performed using the Statistical Package for Social Sciences (SPSS) version 23.0 (Chicago, Illinois, USA). As normality could not be assumed, the central tendency was described as median and interquartile range (IQR), whilst descriptive data was presented as number and frequency. Confirmatory factor analysis (CFA) was performed using Lavaan package in R software for statistical computing and graphics version 3.5.1 (R Foundation, Vienna, Austria) [33].

##### Validity

Face and content validity were verified by an expert panel (which consisted of two primary care physicians and three academic pharmacists as primary care physicians and pharmacists are currently involved in the field of ACP in Malaysia). Flesch reading ease, to test the readability of the instrument was not applied to ACPQ-M because the computer calculated score was not developed and validated for use in Malay language [34].

Factor analysis was used to determine the construct validity. Exploratory factor analysis (EFA) was performed to explore the dimensionality of the ACPQ-M by computing the percentage of total variance explained, the number of factors and factor loadings to determine the degree of agreement between observed scores and latent variables [35]. Bartlett’s test of sphericity was used to test for intercorrelations between all the variables within the correlation matrix [36]. Sampling adequacies were determined using the Kaiser-Meyer-Olkin criterion and communalities of the variables. Kaiser-Meyer-Olkin, factor loading values and communalities of at least 0.6, 0.4 and 0.4, respectively, were deemed as having good construct validity [37].

Confirmatory factor analysis (CFA) was conducted to verify the factor structure of the ACPQ-M [38]. The factor structure was examined by computing the model fit indices such as Comparative Fit Index (CFI), Tucker Lewis Index (TLI); standardised root mean square residual (SRMR) and root mean square error of approximation (RMSEA) using diagonally weighted least square method for categorical variables [35]. CFI and TLI values of at least 0.95 and SRMR value of ≤ 0.09 indicates goodness of fit [35]. Additionally, RMSEA *p*-value of < 0.05 also indicates acceptable level of model fit with the degree of fit being interpreted as such: RMSEA *p-*value < 0.05 close fit, 0.05 to < 0.08 reasonably good, 0.08 to < 0.10 mediocre, and ≥ 0.10 unacceptable [35, 39].

##### Reliability

As with previous psychometric validation of the English ACPQ, Cronbach’s α was used to assess the internal consistency of the items in the ACPQ-M [28]. Cronbach’s α values between 0.70–0.90 implied adequate internal consistency [40]. Corrected item-total correlations were used to identify items which were inconsistent with other items in the ACPQ-M. Corrected item-total correlation values < 0.2 were deemed as unacceptable [40].

Test-retest reliability was assessed using quadratic weighted Cohen’s kappa coefficient as the items for validation were measured as ordinal data on a 5 point Likert-scale [41]. Kappa value of + 1 indicates complete inter-rater agreement for the items, whereas Kappa value of − 1 at the other end of the continuum represents complete disagreement. The agreement level can be further interpreted as follows: < 0 most likely due to chance agreement, 0.01–0.20 slight agreement between inter-rater, 0.21–0.40 fair agreement, 0.41–0.60 moderate agreement, 0.61–0.80 substantial agreement and 0.81–1.00 almost perfect agreement [42].

## Results

A total of 222/232 participants agreed to participate (response rate = 96.0%). The majority were female (72.1%) and Malay (54.1%), with a median age of 29 years (IQR = 12) (Table 1). A total of 200 (90.1%) participants were in favour of ACP.

**Table 1:** Demographic characteristics of participants

Demographic Characteristics	N (%) (*n* = 222)
Female	160 (72.1)
Median age ± interquartile range (years) [range]	29 ± 12 [22–73]
< 40	117 (52.7)
≥ 40	105 (47.3)
Ethnicity	
Malay	120 (54.1)
Chinese	77 (34.6)
Indian	23 (10.4)
Others*	2 (0.9)
Marital status	
Single	132 (59.5)
Married	88 (39.6)
Divorced	1 (0.5)
Widowed	1 (0.5)
Level of education	
Primary (completed 6 years of education)	2 (0.9)
Secondary (completed 12 years of education)	33 (14.9)
Tertiary (completed at least 15 years of education)	187 (84.2)
Religion	
Islam	120 (54.0)
Buddhism	60 (27.0)
Christianity	21 (9.5)
Hinduism	20 (9.0)
Others**	1 (0.5)
Occupation	
Employed	170 (76.6)
Unemployed	52 (23.4)
Personal monthly income	
< RM1000 ($250)	51 (23.0)
RM1001-RM2000 ($251–$500)	49 (22.1)
RM2001-RM3000 ($501–$750)	51 (23.0)
RM3001-RM4000 ($751–$1000)	30 (13.5)
RM4001-RM5000 ($1001–$1250)	9 (4.1)
> RM5001 ($1251)	32 (18.9)
Living companions	
Family (spouse, siblings, children)	160 (72.1)
Friends	42 (18.9)
Alone	20 (9.0)
Self-rated health status	
Excellent	27 (12.2)
Very good	79 (35.6)
Good	108 (48.6)
Poor	8 (3.6)
Presence of co-morbidities	52 (25.8)
Asthma / Chronic obstructive pulmonary disease	12 (5.0)
Hypertension	11 (4.8)
Diabetes mellitus	10 (4.4)

* Murut (*n* = 1), Punjabis (*n* = 1); ** Sikhism (*n* = 1)

### Validity

EFA found that the ACPQ-M was a 4-factor model, which explained > 50% of the total variance of each domain (Table 2). Kaiser-Meyer-Olkin and factor loadings were > 0.6 and > 0.4, respectively. Communalities were > 0.4 except for the item “I believed that the discussion of the topic of death was seen as “unlucky” and I tried to avoid discussing about it”. Bartlett’s tests of sphericity were significant for all domains (*p* < 0.05).

**Table 2:** Exploratory factor analysis of the Malay Advance Care Planning Questionnaire (ACPQ-M)

Domains [no. of items]	Items	Factor loadings				Total variance explained (%)	Communalities	Keiser-Meir-Olkin	Barrett’s Test of Sphericity	
		1	2	3	4				Chi-square	*p-*value
Feelings regarding advance care planning [5] (*n* = 222)	Felt better to have expressed wished in advance if I had a heart attack or on a breathing machine	0.931				82.6	0.867	0.879	1075.0	< 0.001
	Felt better to have expressed wished in advance if I had severe dementia	0.916					0.839			
	Felt better to have expressed wished in advance if I had a stroke	0.913					0.833			
	Felt better to have expressed wished in advance if I had a road accident or in a coma	0.893					0.798			
	Felt better to have expressed wished in advance if I had cancer	0.889					0.790			
Justifications for advance care planning [4] (*n* = 200)	I am aware that I could possibly lose my decision-making power as a result of becoming seriously ill or injured		0.779			52.0	0.607	0.704	131.0	< 0.001
	I want to be able to make my own decision		0.710				0.505			
	I hope to not burden my family with my medical treatment preferences		0.705				0.497			
	There may be differences in opinions between my family members		0.685				0.470			
Justifications for not having advance care planning: Fate and religion [4] (*n* = 22)	I believed that planning for my death would mean there is no hope for me			0.851		51.5	0.724	0.639	14.1	0.028
	I will take it as it comes, as I have no control over my death			0.780			0.609			
	I felt that it was best to leave to fate or to God			0.727			0.529			
	I believed that the discussion of the topic of death was seen as “unlucky” and I tried to avoid discussing about it			0.446			0.199			
Justifications for not having advance care planning: Avoid thinking about death [3] (*n* = 22)	I do not want to think I will die or lose my memory				0.912	78.8	0.832	0.725	26.7	< 0.001
	I cannot imagine myself in such a situation				0.879		0.772			
	I am currently healthy and there is no need to consider such decisions				0.871		0.759			

CFA found that the CFI values for the four domains ranged from 0.985–1.000; while the TLI values ranged from 0.956–1.000. The SRMR values for the domains “feelings regarding ACP”, “justifications for ACP” and “justifications for not having ACP: fate and religion” were 0.021, 0.047 and 0.062, respectively. The *p*-value of RMSEA for the domains “feelings regarding ACP”, “justifications for ACP” and “justifications for not having ACP: fate and religion” were 0.088, 0.045 and 0.339, respectively. SRMR and RMSEA for the domain “justifications for not having ACP: avoid thinking about death” were unable to be calculated probably due to inadequate data point or number of participants (df = 0, *n* = 8). RMSEA for the domain “justifications for not having ACP: fate and religion” did not suggest good fit probably due to the similar reason of inadequate data point or number of participants (*n* = 8).

### Reliability

The Cronbach’s α values for the four domains ranged from 0.674–0.947 (Table 3). The corrected item total correlation of all items was > 0.2. The deletion of item ‘I believed that the discussion of the topic of death was seen as “unlucky” and I tried to avoid discussing about it’ in the domain “justifications for not having ACP: fate and religion” would increase the Cronbach’s α from 0.674 to 0.725.

**Table 3:** Reliability of the Malay Advance Care Planning Questionnaire (ACPQ-M)

New domains (no. of items)	Items	Cronbach alpha	Corrected item total correlation	Cronbach alpha if item deleted	Cohen’s kappa (quadratic weighted)
Feelings regarding advance care planning [5] (*n* = 157)	Felt better to have expressed wished in advance if I had a heart attack or on a breathing machine	0.947	0.890	0.928	0.613
	Felt better to have expressed wished in advance if I had severe dementia		0.866	0.932	0.639
	Felt better to have expressed wished in advance if I had a stroke		0.860	0.933	0.622
	Felt better to have expressed wished in advance if I had a road accident or in a coma		0.833	0.939	0.674
	Felt better to have expressed wished in advance if I had cancer		0.825	0.939	0.465
Justifications for advance care planning [4] (*n* = 149)	I am aware that I could possibly lose my decision-making power as a result of becoming seriously ill or injured	0.688	0.536	0.556	0.444
	I want to be able to make my own decision		0.469	0.619	0.419
	I hope to not burden my family with my medical treatment preferences		0.447	0.514	0.477
	There may be differences in opinions between my family members		0.440	0.651	0.340
Justifications for not having advance care planning: Fate and religion [4] (*n* = 8)	I believed that planning for my death would mean there is no hope for me	0.674	0.644	0.480	0.100
	I will take it as it comes, as I have no control over my death		0.535	0.551	−0.043
	I felt that it was best to leave to fate or to God		0.448	0.622	0.467
	I believed that the discussion of the topic of death was seen as “unlucky” and I tried to avoid discussing about it		0.228	0.725	0.000
Justifications for not having advance care planning: Avoid thinking about death [3] (*n* = 8)	I do not want to think I will die or lose my memory	0.862	0.788	0.761	0.333
	I cannot imagine myself in such a situation		0.724	0.823	−0.200
	I am currently healthy and there is no need to consider such decisions		0.716	0.836	0.000

Only 157/222 participants completed retest (response rate = 71%), as the remaining participants were either uncontactable (*n* = 7) or refused to participate at retest (*n* = 58). At test-retest, quadratic weighted kappa values for all domains ranged from 0.340–0.674; except for the domains “justifications for not having ACP: fate and religion” and “justifications for not having ACP: avoid thinking about death” which ranged from −0.200-0.467.

## Discussion

The ACPQ-M was found to be a valid and reliable instrument to assess the KAP regarding ACP in Malaysia. The final version of the ACPQ-M consists of 4 sections and 60 items, of which 29 were measured on a nominal scale whilst 31 items were measured on a 5-point Likert scale.

Study on dimensionality using EFA showed that the ACPQ-M was a 4-factor model and a good model of fit. Our findings was similar to the English ACPQ [28]. The four domains were “feelings regarding ACP”, “justifications for ACP”, “justifications for not having ACP: avoid thinking about death” and “justifications for not having ACP: fate and religion”. There was adequate sampling in all domains (Kaiser-Meyer-Olkin > 0.6). Bartlett’s tests of sphericity were also significant for all domains which implied that the correlations between items were sufficient for factor analysis. Additionally, adequate amount of the variance can be explained by the underlying factors as indicated by the communalities > 0.4 except for the item “I believed that the discussion of the topic of death was seen as unlucky and I tried to avoid discussing about it”. However, the factor loading for this item was > 0.4. Hence, this item was retained as patients were able to understand the questionnaire without issue during validation of the English ACPQ, and we wanted the two versions to be the same [28]. We were unable to compare our findings with any other studies as the previous studies involving validation of the KAP instrument did not perform EFA [14, 15].

Overall, the reliability of the ACPQ-M was satisfactory (ranged between 0.674–0.947). This was consistent with the reliability of English ACPQ [28] and comparable to a previous KAP study conducted on younger adults (0.76–0.93) [14]. However, the test-retest reliability computed using quadratic weighted kappa was low for two domains: “Justifications for not having ACP: fate and religion” and “Justifications for not having ACP: avoid thinking about death”. We believed this to be attributed to the small number of participants (*n* = 8) who were not in favour of advance care planning. The kappa values for these two domains of the ACPQ-M were also lower than the English ACPQ as more participants who were not in favour of ACP answered the English ACPQ (*n* = 55) as opposed to the ACPQ-M (*n* = 8) [28]. In addition, the English ACPQ study recruited older participants (median age = 68 years) when compared to the present study (median age = 29 years) [28]. The proportion of participants who favoured ACP was significantly higher than the previous study [28] could be due to the higher number of the young participants. A prior study has reported that younger adults tend to have more favourable attitudes toward ACP [14]. Similarly, we were unable to compare our findings with any other studies as the previous study which developed and validated the KAP instrument did not perform test-retest analysis [14, 15].

As decision making in ACP involved a complicated process [19], various instruments have been developed to measure the different aspects of ACP. Some instruments have been developed to elicit information regarding attitude towards patient autonomy [15] and to examine the difference in influence of cultural values and beliefs among African and White Americans towards ACP [16]. Other instruments were developed and validated to evaluate behaviour change as a result of an ACP intervention [17–19, 43] and to assess the role of surrogate decision makers of patients with chronic illness in ACP [44]. Most of the validated instruments were developed in Korean [15], English [14, 16, 17, 19], French [45] or Spanish languages [46]. To date, there is a lack of validated instruments that assessed the KAP of adults regarding ACP. Only one instrument that assessed the KAP among younger adults has been developed in the United States [14].

A study that assesses the KAP is needed to determine readiness of a population for advance care planning. Hence, it was necessary to translate and validate the ACPQ-M as Malay is the national language of Malaysia, so that Malaysians who speak Malay are able to answer the questionnaire in addition to the previously validated English ACPQ.

### Limitations of the study

One of the limitations of the study was that convergent and discriminative validity were not performed. Convergent validity was not performed as there were no other validated instruments in Malay to assess the knowledge, attitude and practice during the period of our study. Discriminative validity was also not performed as it was not possible to identify participants who had high or low knowledge of ACP. In addition, a higher number of younger participants were recruited by chance due to convenience sampling. Younger adults (≥21 years) were recruited in this study as the subsequent phase of this study will assess the readiness of community dwelling adults regarding ACP. The inclusion of a younger age group may have the advantage of improving the validity and reliability of the ACPQ-M as this tool can be used to inform the readiness of community dwelling adults regarding ACP. However, the limitation is that our results may not be comparable to previous studies. Thirdly, it was not possible for us to conduct the retest “face to face” as participants were reluctant to come back to the hospital just to answer a questionnaire. However, the same researcher who recruited participants at baseline, re-administered the questionnaire again over the phone.

### Strength of the study

Although cross cultural adaptation was not performed, this manuscript has described the validation process and the psychometric properties of the ACPQ-M. Despite the limitations, this study has validated the ACPQ-M and found that this tool had adequate psychometric properties. The tool can now be used to assess the KAP regarding ACP among Malaysians who are only able to understand Malay. Findings from the administration of this tool can assist policymakers to decide if Malaysians are ready for ACP to be legislated in Malaysia.

## Conclusion

The ACPQ-M was found to be a 4-factor model, and a valid and reliable instrument to assess the KAP regarding ACP in Malaysia. This instrument can contribute to profound understanding of the KAP of Malaysians regarding ACP, and assist policy makers in determining the readiness for legislation of ACP in Malaysia.

## Data Availability

The datasets used and/or analysed during the current study are available from the corresponding author on reasonable request.

## Abbreviations

ACP: Advance care planning
ACPQ: Advance care planning questionnaire
ACPQ-M: Malay Advance care planning questionnaire
CFA: Confimatory factor analysis
CFI: Comparative Fit Index
EFA: Exploratory factor analysis
IQR: Inter-quartile range
KAP: Knowledge, attitude and practice
RMSEA: Root Mean Square Error of Approximation
SRMR: Standardised Root Mean Square Residual
TLI: Tucker Lewis Index
